# Digital Tools to Facilitate the Detection and Treatment of Bipolar Disorder: Key Developments and Future Directions

**DOI:** 10.2196/58631

**Published:** 2024-04-01

**Authors:** Taiane de Azevedo Cardoso, Shruti Kochhar, John Torous, Emma Morton

**Affiliations:** 1 The Institute for Mental and Physical Health and Clinical Translation School of Medicine Deakin University Geelong Australia; 2 JMIR Publications Toronto, ON Canada; 3 Digital Psychiatry Department of Psychiatry Beth Israel Deaconess Medical Center Boston, MA United States; 4 School of Psychological Sciences Monash University Clayton Australia

**Keywords:** bipolar disorder, digital phenotyping, machine learning, mobile health interventions, mobile health, mHealth, apps

## Abstract

Bipolar disorder (BD) impacts over 40 million people around the world, often manifesting in early adulthood and substantially impacting the quality of life and functioning of individuals. Although early interventions are associated with a better prognosis, the early detection of BD is challenging given the high degree of similarity with other psychiatric conditions, including major depressive disorder, which corroborates the high rates of misdiagnosis. Further, BD has a chronic, relapsing course, and the majority of patients will go on to experience mood relapses despite pharmacological treatment. Digital technologies present promising results to augment early detection of symptoms and enhance BD treatment. In this editorial, we will discuss current findings on the use of digital technologies in the field of BD, while debating the challenges associated with their implementation in clinical practice and the future directions.

## Introduction

Bipolar disorder (BD) is a chronic and recurrent mental illness that affects 2.4% of the worldwide population [1]. BD usually manifests in early adulthood, with the median age at onset found to be 33 years of age and a peak age at onset of 19.5 years of age [2]. BD presents a profound negative impact on individuals’ lives with high rates of disability [3]. According to the Global Burden of Disease Study (2019), BD is the 12th leading cause of years lived with disability among young adults aged between 15 to 24 years [4].

**Figure 1:**
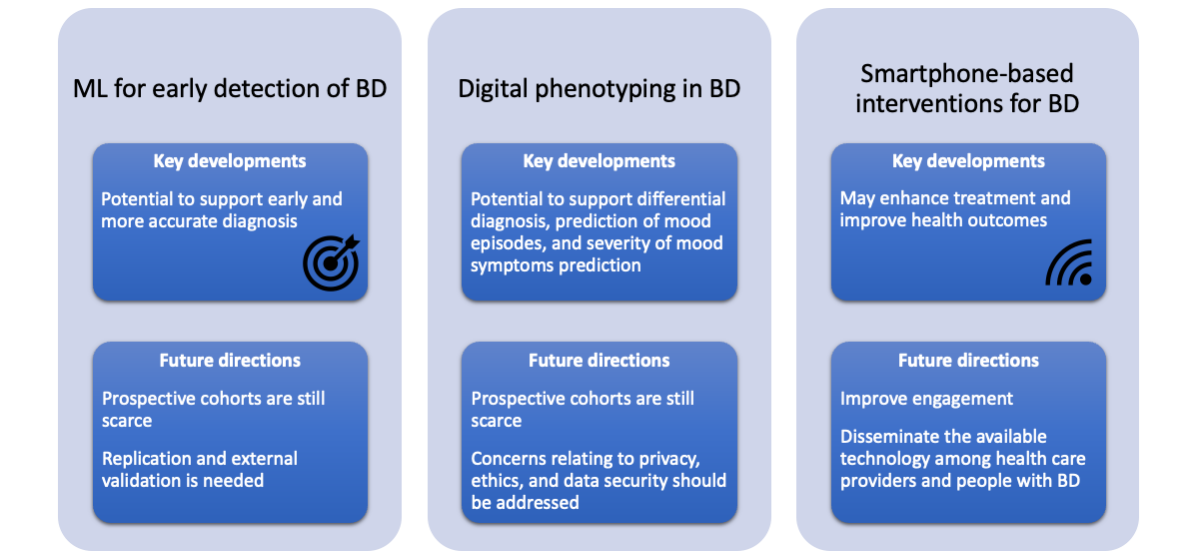
Digital tools for BD: key developments and future directions. BD: bipolar disorder; ML: machine learning.

Digital health technologies have been studied in the context of BD and are showing promising results in the early detection of the disorder [5,6] and depressive or manic episodes among individuals with the disorder [7], as well as the promotion of a better prognosis [8,9]. To understand this progress, we will review 3 promising and innovative areas of work (Figure 1). In this editorial, we will discuss the role of (1) machine learning techniques, (2) digital phenotyping, and (3) mobile health (mHealth) apps to enhance BD care. Additionally, the challenges and future directions for the implementation of digital health technologies in BD will be considered.

## Early Detection of BD and Reducing Misdiagnosis: Insights From Machine Learning Studies

A potential contributor to the disease burden in BD is the delay in obtaining an accurate diagnosis, which consequently delays the appropriate management and treatment of the disorder. A recent systematic review showed that the median delay in help seeking was 3.5 years, the median delay in diagnosis was 6.7 years, and the median duration of untreated BD was 5.9 years [10]. Another recent study found that the rate of misdiagnosis in BD was 76.8%, and most of those cases received a misdiagnosis of major depressive disorder (MDD) [11]. Despite the similarities in the clinical presentation of a depressive episode in MDD and BD, the treatment strategies recommended for each disorder are different, with antidepressants being the main pharmacological strategy in MDD [12] and mood stabilizers being recommended for BD [13]. Thus, having strategies for the early detection of BD is crucial to reduce the misdiagnosis rates and to provide the proper treatment early in the course of the disorder. In this section, we will be describing some machine learning studies aimed at (1) predicting mood disorder misdiagnosis, (2) predicting BD onset, and (3) differentiating BD from unipolar disorder. Finally, we will discuss the challenges of translating these findings into clinical practice.

A scoping review aimed at investigating the use of machine learning techniques for the detection of BD found that the majority of the studies used classification models (eg, random forest), included a sample size of fewer than 300 individuals, and included clinical data in the model [5]. The potential of new machine learning methods to better understand factors associated with misdiagnosis was exemplified in a recent study that reported a misdiagnosis rate of 50.97% [14]. In this study, any mismatch between the self-reported diagnosis and the clinical interview diagnosis was considered a misdiagnosis. The investigators used machine learning techniques to identify the predictors of misdiagnosis, and the mean accuracy of the predictive model was 70% [14]. This study showed that more severe depressive symptoms and unstable self-image were the strongest predictors of mood disorder misdiagnosis among the 1045 variables evaluated [14]. These results may be explained by the fact that patients usually seek treatment when they are severely depressed and that they may be underreporting hypomanic symptoms during a severe depressive episode. Consequently, they might be misdiagnosed with major depression instead of receiving the correct diagnosis of BD.

Another recent study highlights how a correct diagnosis may be made earlier. The clinical predictors of BD were described in a large birth cohort study, including 3748 subjects assessed at birth and 11, 15, 18, and 22 years of age [6]. The study used machine learning techniques and showed that the presence of suicide risk, generalized anxiety disorder, parental physical abuse, and financial problems at 18 years of age were the strongest predictors for a BD diagnosis at 22 years of age, with a balanced accuracy of 75% [6]. Additionally, the high-risk subgroup of BD showed a high frequency of drug use and depressive symptoms [6].

Several machine learning studies used digital phenotyping to classify BD and unipolar disorder [15,16]. In one study, daily smartphone-based self-assessments of mood and same-time passively collected smartphone data on smartphone usage were assessed for 6 months [15]. The main findings indicate that patients with BD, in an euthymic state, had a lower number of incoming phone calls per day compared to patients with unipolar depression also experiencing euthymia. In addition, during depressive states, patients with BD had a lower number of incoming and outgoing phone calls per day as compared with patients with unipolar depression [15]. BD was classified with an area under the curve (AUC) of 0.84 (overall; when mood state was not taken into consideration), 0.86 (during a depressive state), and 0.87 (during a euthymic state) in this study. However, when applying the leave-one-out cross-validation approach, the AUC for all models reduced (AUC=0.48 for the overall model, AUC=0.42 for the depressive state model, and AUC=0.46 for the euthymic state model), indicating that changes in combined smartphone-based data were highly individual [15]. Another digital phenotyping study using the mindLAMP app to collect geolocation, accelerometer, and screen/state reported an AUC of 0.62 for classifying patients with MDD or Bipolar I/II disorders [16]

The differing results noted above are common in machine learning research, especially where the underlying data and technology differ between studies. A task force discussing the scientific literature related to machine learning and big data–based studies showed that machine learning studies have included a variety of data to predict BD, including neuroimaging, genetics, electroencephalogram, neurophysiological data, blood biomarkers, text, facial expressions, speech, and ecological momentary assessments [17]. The task force emphasized that some limitations should be addressed to allow these findings to be translated to clinical practice, in particular the lack of external validation of the predictive models [17].

## Digital Phenotyping to Detect Mood Symptoms and Mood Episodes in BD

The development of digital phenotyping is quickly evolving and expanding in the field of BD. Digital phenotyping involves collecting data (eg, location, activity, sleep, speech patterns), typically from smartphones, to monitor behavior, cognition, and mood [18]. Digital phenotyping may help facilitate the early detection of potentially problematic mood changes, therefore facilitating early intervention. Before digital phenotyping can be applied in usual care for BD, we must develop an understanding of which of the multitude of digital data collected by smartphones and wearable sensors can reliably and validly detect early warning signs of mood episodes. Importantly, while several studies have shown that digital phenotyping is a promising technique, it faces several challenges that need to be robustly addressed [19], which will be discussed in this section.

A systematic review describing the evidence about the use of portable digital tools for detecting BD, mood states, and mood symptoms found 62 studies assessing it in terms of four main areas: (1) smartphone apps designed to collect active (eg, mood self-assessments) or passive (eg, recording geolocation, step counts, call and text logs, sleep, etc) data; (2) wearable sensors for the monitoring of electrocardiography and actigraphy; (3) audio-visual recordings for the analysis of speech or facial expressions and upper body movements; and (4) multimodal tools, combining 2 or more of the above [7]. Two-thirds of the studies included applied machine learning approaches to classify BD versus healthy controls, to identify mood states, or to predict the severity of symptoms. They achieved mixed results, yielding fair to excellent classification performances, with accuracy globally ranging from 60% to 97% [7]. A recent review assessing the application of digital tools for major depressive episodes described the following digital phenotype for BD: (1) speech alterations during a depressive episode, including decreased speech pause and reduced fundamental frequency, while these speech features were increased during a hypomanic episode; (2) irrespective of the mood state, heart rate variability was reduced, but the change in heart rate variability in the interepisodic phases remained unclear, and (3) an electrodermal hypoactivity in a depressive episode was reported, which increased when patients were euthymic [20].

Regarding the challenges related to digital phenotyping, it is important to note that any data collected using digital devices is prone to bias and needs to be standardized to ensure accuracy not only across populations but also across different devices. Moreover, concerns relating to privacy, ethics, data security, and consent must be addressed. User comfort in sharing data differs depending on the data type (eg, users are more comfortable sharing health data than personal data such as location, communication logs, and social activity) and the recipient (eg, users have greater comfort sharing data directly with clinicians than having this entered into their electronic health record), and this may impact willingness to use digital phenotyping platforms [21]. As user engagement is essential for the success of any digital phenotyping tools [22], it is necessary to account for discrepancies in access, equity, and distribution of resources. Finally, more in-depth longitudinal studies are required to ascertain the relationship between biomarkers and long-term outcomes of health and well-being.

## Smartphone-Based Interventions for BD

Psychosocial therapies and education in self-management strategies can improve outcomes in BD [23] and are recommended complements to pharmacological interventions in guidelines for BD treatment. However, access to these forms of care remains suboptimal, with less than 50% of individuals in treatment for BD receiving therapy with a psychologist, social worker, or self-help support group [24]. Smartphone apps have the potential to provide psychoeducation and facilitate several of the core components of psychosocial therapies (eg, self-monitoring, detecting and responding to mood episodes, stabilizing daily routines, improving emotion regulation, encouraging medication adherence, etc) [25]. Encouragingly, individuals with BD report high levels of access to smartphones and a willingness to receive psychosocial interventions via apps [26,27]. Several app-facilitated interventions have been developed and evaluated for BD, variously integrating self-monitoring, psychoeducation, cognitive-behavior therapy, and skills training, and targeting both symptoms and patient-valued outcomes such as functioning and quality of life [9]. However, evidence for their feasibility and efficacy is still preliminary, and interventions are yet to fully leverage the capabilities of apps for intervention personalization.

Two recent systematic reviews and meta-analyses investigated the role of smartphone-based interventions to improve clinical outcomes in BD and found conflicting results [8,9]. Liu et al [8] included 10 studies in their systematic review (7 randomized controlled trials and 3 single-arm trials) and concluded that smartphone-based interventions were effective in reducing manic and depressive symptoms in between-group (compared to controls) and within-group (comparing symptoms from baseline to postintervention in the intervention group). Anmella et al [9] included 13 studies in their qualitative synthesis of the findings and 5 studies in their meta-analysis. The meta-analyses comparing the pre-post change in depressive and (hypo)manic symptom severity, functioning, quality of life, and perceived stress between smartphone interventions and controls did not reach statistical significance for any outcome assessed [9]. The potential explanation for the conflicting findings is that the eligibility criteria were different between both studies. The most important difference is the fact that Liu et al [8] included not only smartphone-based apps but also phone calls from specialists to facilitate therapy and website interventions in the intervention group, while Anmella et al [9] excluded interventions not delivered through smartphones (eg, exclusive of phone calls, phone messaging, only SMS text messaging, or computer-delivered interventions) from the intervention group. Another difference between both studies is that Liu et al [8] did not restrict the inclusion criteria to individuals with BD and included a few studies that recruited a more heterogeneous population (eg, serious mental illness, mood disorders), while Anmella et al [9] only included studies where the participants were diagnosed with BD.

Given the heterogeneity of BD both between and within individuals, effective psychotherapy involves appropriately tailoring intervention content and delivery to the challenges and goals of a specific individual at a specific time. However, apps are yet to fully capitalize on the potential of smartphones to personalize intervention delivery in response to changes in clinical state. One app program, SIMPLe, personalizes content using ecological momentary assessment to identify potential prodromal mood changes and adapts the delivery of psychoeducation messages in response [28,29]. To advance our understanding of how to tailor just-in-time adaptive interventions for BD, microrandomized trials can be used to evaluate the immediate impact of diverse types of intervention prompts. For example, an evaluation of mobile acceptance and commitment therapy used this trial design to evaluate different categories of intervention and found that awareness-focused prompts paradoxically increased symptoms [30]. Beyond the clinical utility of personalization, this feature is also highly prioritized by individuals with BD themselves [31], who have expressed a desire for apps that make meaningful use of their data to customize intervention delivery and facilitate proactive support.

## Improving the Dissemination and Uptake of Apps for BD

Although research-led studies have developed and evaluated mobile apps for BD, the dissemination and uptake of these apps in real-world contexts must be considered to reach the target population and maximize their impact. A recent web-based survey investigating the use of mobile apps to support mood and sleep self-management among individuals with BD found that 41.6% of participants reported using a self-management app related to mood and/or sleep [32]. The most nominated app for mood monitoring was Daylio, and the most reported app for sleep monitoring was Fitbit. Since these apps are designed to support the public with well-being concerns, this raises questions about why apps specifically designed for BD are not reaching this population. Two possibilities emerge: (1) apps designed for BD are not sufficiently acceptable or engaging in the eyes of the target audience and (2) individuals with BD may not be adequately supported to select the app that is best suited to their needs. To facilitate research-led apps reaching and impacting users with BD, we must consider their ability to create and sustain user engagement. Further, we must consider effective dissemination pathways, targeting both patients and the health care providers involved in the provision of care to people with BD (eg, clinicians, nurses, allied health professionals, and case managers).

**Figure 2:**
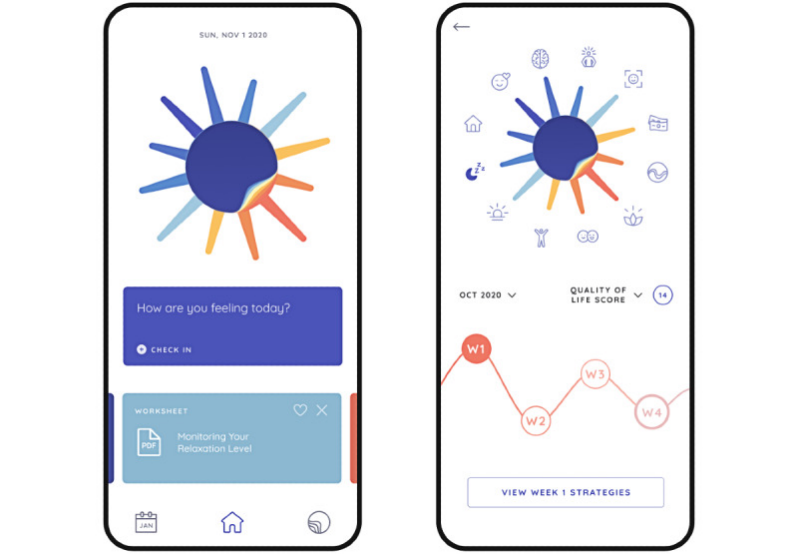
Interface of the PolarUs app (reproduced from Michalak et al [37], which is published under Creative Commons Attribution 4.0 International License [43]).

Poor engagement is endemic to mental health apps in general, extending beyond just those aimed at BD [33], with most users of publicly available apps disengaging within 30 days. Specific to BD, a systematic review showed that adherence data in research trials was infrequently reported; of the 13 studies providing engagement data, the activity rate ranged from 58% to 91% [34]. The failure to consider the needs and goals of the targeted population likely contributes to startlingly poor levels of uptake and adherence. Involving users in the design of apps can help ensure their design, content, and feature selection are relevant, acceptable, and engaging. However, a recent review investigated the level of user involvement in the design of self-monitoring apps for BD [35] and found that 36% of the apps did not mention user involvement in the design, while 9% reported low, 36% reported medium, and 18% reported high user involvement. This review highlights the importance of including an appropriate sample size capable of adequately capturing users’ needs so that technology can be better designed. Finally, it is recommended that users are involved early in the design process, and their involvement should not be limited solely to the design but also to all aspects of the research, ensuring end-to-end involvement. Case studies of apps using a co-design framework include the quality of life–focused LiveWell and PolarUs apps, both of which consulted people with BD throughout development [36,37]. Figure 2 depicts 2 screens from the PolarUs app: on the home screen (left image), users are prompted to engage in quality of life, sleep, and mood self-monitoring, and are provided with relevant resources [37]. Users can review their self-monitoring data over time (right image). Individuals with BD provided input into the app design (including icons, color scheme, and layout), navigation, features, and content.

**Figure 3:**
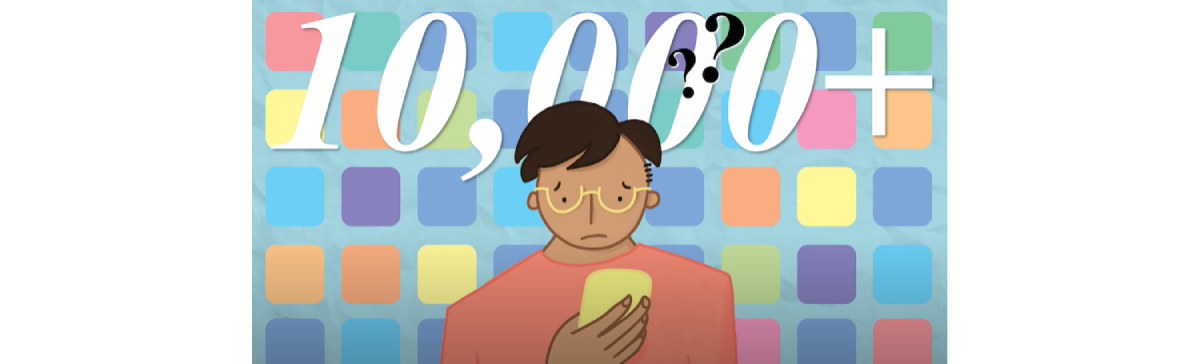
Choosing a bipolar disorder app that works for you (reproduced from [40], with permission from Erin Michalak).

**Figure 4:**
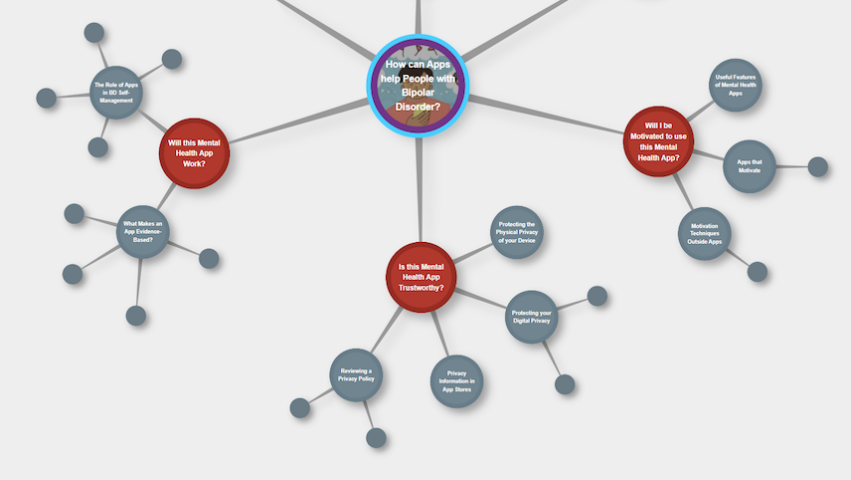
Additional information and resources to support people in evaluating app privacy policies, inclusion of evidence-based strategies for BD, and motivational techniques (reproduced from [41], with permission from Erin Michalak). BD: bipolar disorder.

Looking ahead, as more apps for BD are developed and made available to the public, patients with BD and health care providers will likely require support to navigate the digital health landscape, as research-led apps will compete for attention with commercial offerings that may have limitations in their privacy protections and efficacy [38]. Educational interventions to enhance digital health literacy may help individuals with BD to select the appropriate apps for their self-management goals. While in general, levels of digital health literacy are comparable for people with BD to the general population, a study found that individuals with BD who are younger, have completed less education, or are less familiar with mental health apps may require extra support to safely and productively navigate web-based health resources [39]. Recent steps have been taken to address the needs of these groups: a brief, informational video describing strategies to select safe, effective, and engaging mental health apps for BD was created [40], incorporating the perspectives of people with lived experience in the script and design. A still image from this video is presented in Figure 3 [40]. This resource was later expanded upon to create a web-based module [41], depicted in Figure 4, which contains additional information and resources to support people in evaluating app privacy policies, inclusion of evidence-based strategies for BD, and motivational techniques. Other resources like mindapps.org can help facilitate informed decision-making about mental health apps [38].

Health care providers are an important source of information and advice on smartphone apps, yet a web survey found that only 48.8% of health care providers reported discussing or recommending health apps to patients with BD [42]. Most of the apps recommended were related to core symptoms of BD, including mood and sleep. Among the health care providers who did not discuss health apps with patients with BD (51.2%), the predominant reason mentioned was the lack of familiarity with credible and suitable apps tailored for BD. The resources discussed above are also appropriate for use by clinicians wishing to learn more about appropriate and effective apps for BD [38,41]. These findings emphasize the importance of providing training aimed at increasing clinician self-efficacy in using mobile apps with patients, a strategy that should be considered by researchers developing new mHealth tools.

## Conclusion

The evidence available to date indicates that digital technologies may help in the early detection of BD and mood episodes, as well as in enhancing treatment, improving health outcomes and consequently promoting a better prognosis for individuals with BD. However, there are important limitations that need to be addressed before these technologies can be translated to clinical practice, including the following: (1) external validation of the machine learning models developed to date, (2) need for well-designed prospective cohort studies to validate findings about digital phenotyping and early detection of BD and mood symptoms, (3) involvement of individuals with lived experience in the development of mobile apps, and (4) dissemination of the available technology among health care providers and directly to people with BD. Finally, adequately powered randomized controlled trials are still needed to evaluate the efficacy of mental health apps for BD. Additionally, there is a need to advance our understanding of how to tailor app-based interventions based on the valuable insights generated by digital phenotyping.

## Abbreviations

AUC: area under the curve
BD: bipolar disorder
MDD: major depressive disorder
mHealth: mobile health
